# Increased likelihood of heat-induced large wildfires in the Mediterranean Basin

**DOI:** 10.1038/s41598-020-70069-z

**Published:** 2020-08-14

**Authors:** Julien Ruffault, Thomas Curt, Vincent Moron, Ricardo M. Trigo, Florent Mouillot, Nikos Koutsias, François Pimont, Nicolas Martin-StPaul, Renaud Barbero, Jean-Luc Dupuy, Ana Russo, Chiraz Belhadj-Khedher

**Affiliations:** 1 Institut Méditerranéen de Biodiversité et d’Ecologie marine et continentale (IMBE), Aix Marseille Université, CNRS, IRD, Avignon Université, Avignon, France; 2 INRAE, Aix Marseille Univ, RECOVER, Aix-en-Provence, France; 3 Aix Marseille University, CNRS, IRD, INRAE, Coll. de France, CEREGE, Aix-en-Provence, France; 4 Instituto Dom Luiz (IDL), Faculdade de Ciências, Universidade de Lisboa, Campo Grande, 1749-016 Lisbon, Portugal; 5 CEFE, UMR 5175, Univ. Montpellier, CNRS, EPHE, IRD, univ. Paul Valery Montpellier 3, 1919 route de Mende, 34293 Montpellier Cedex 5, France; 6 Department of Environmental Engineering, University of Patras, G. Seferi 2, 30100 Agrinio, Greece; 7 INRAE, Ecologie des Forêts Méditerranéennes (UR 629), Avignon, France; 8 Geomatics and Geosystems, University of Manouba, Manouba, Tunisia; 9 Present Address: INRAE, Ecologie des Forêts Méditerranéennes (UR 629), Avignon, France; 10 Departamento de Meteorologia, Instituto de Geociências, Universidade Federal do Rio de Janeiro, Rio de Janeiro, 21941-916 Brazil

**Keywords:** Natural hazards, Climate-change impacts, Ecology, Fire ecology

## Abstract

Wildfire activity is expected to increase across the Mediterranean Basin because of climate change. However, the effects of future climate change on the combinations of atmospheric conditions that promote wildfire activity remain largely unknown. Using a fire-weather based classification of wildfires, we show that future climate scenarios point to an increase in the frequency of two heat-induced fire-weather types that have been related to the largest wildfires in recent years. Heat-induced fire-weather types are characterized by compound dry and warm conditions occurring during summer heatwaves, either under moderate (*heatwave* type) or intense (*hot drought* type) drought. The frequency of heat-induced fire-weather is projected to increase by 14% by the end of the century (2071–2100) under the RCP4.5 scenario, and by 30% under the RCP8.5, suggesting that the frequency and extent of large wildfires will increase throughout the Mediterranean Basin.

## Introduction

Wildfire is a complex phenomenon that occurs when three conditions are met: available fuel, an ignition source (due to lightning or human activities) and weather conditions conducive to fires (fire weather)^1^. Climate and weather are important drivers of wildfire activity across a range of timescales^2–5^, and, consequently, current and potential future climate-induced changes in wildfire activity might threaten ecosystems and human well-being^6^. In most Euro-Mediterranean countries, wildfire activity has been declining owing to management and suppression measures undertaken since the 1980s^7^. However, some recent extreme wildfire events, including those that occurred in 2016 in France^8^, 2017 in Spain and Portugal^9^ and 2018 in Greece^10^ have highlighted the limits of wildfire suppression capabilities under exceptional fire-weather conditions. Furthermore, studies show that wildfire activity is expected to increase across the Mediterranean Basin due to climate change^11,12^. However, how the combinations of climate and weather conditions that promote the largest wildfires will respond to climate change remain largely unknown.

Climate and weather are both drivers of wildfire activity. Soil moisture deficit over days to months increases fuel aridity and flammability^6,13^ while a number of synoptic weather conditions associated to different combinations of short-term and instantaneous meteorological fields (precipitation, temperature, relative humidity and wind speed) influence wildfire behavior^5,14^. Most of the largest wildfires occur when these conditions are met^8^. For instance, the combination of extreme drought with extreme wind or heatwaves have both been identified as crucial factors in the occurrence of crown wildfires in Mediterranean forests and shrublands^15–17^.

In this study, we focus on the frequency of current and future weather and climate conditions associated with wildfires in four countries (France, Portugal, Greece, Tunisia) of the Mediterranean basin covering most of its diverse biogeographic and climatic conditions (Supplementary Figs. S1–S3). Building on the insights gained from a series of previous studies^8,17–19^, we decompose “fire-weather” into robust and distinct combinations of local-scale short-term weather (i.e. daily mean temperature, relative humidity and wind speed) and long-term climate (i.e. monthly to seasonal drought) conditions called fire-weather types (FWTs). First, to identify FWTs, we classify the local-scale weather and climate conditions associated with more than 17,000 records of wildfires (wildfires larger than 30 ha) using an objective clustering. Next, we evaluate whether the largest wildfires (from 80 up to 2,150 ha fire size thresholds) occur preferentially under specific FWTs. Then, we extrapolate this wildfire-based classification to the whole Mediterranean Basin to assess the potential current and future changes to FWT frequency over the twenty-first century. Finally, we discuss the potential consequences in terms of future wildfire activity across the Mediterranean Basin.

## Results

### Identification and characterization of fire weather types (FWTs)

**Figure 1:**
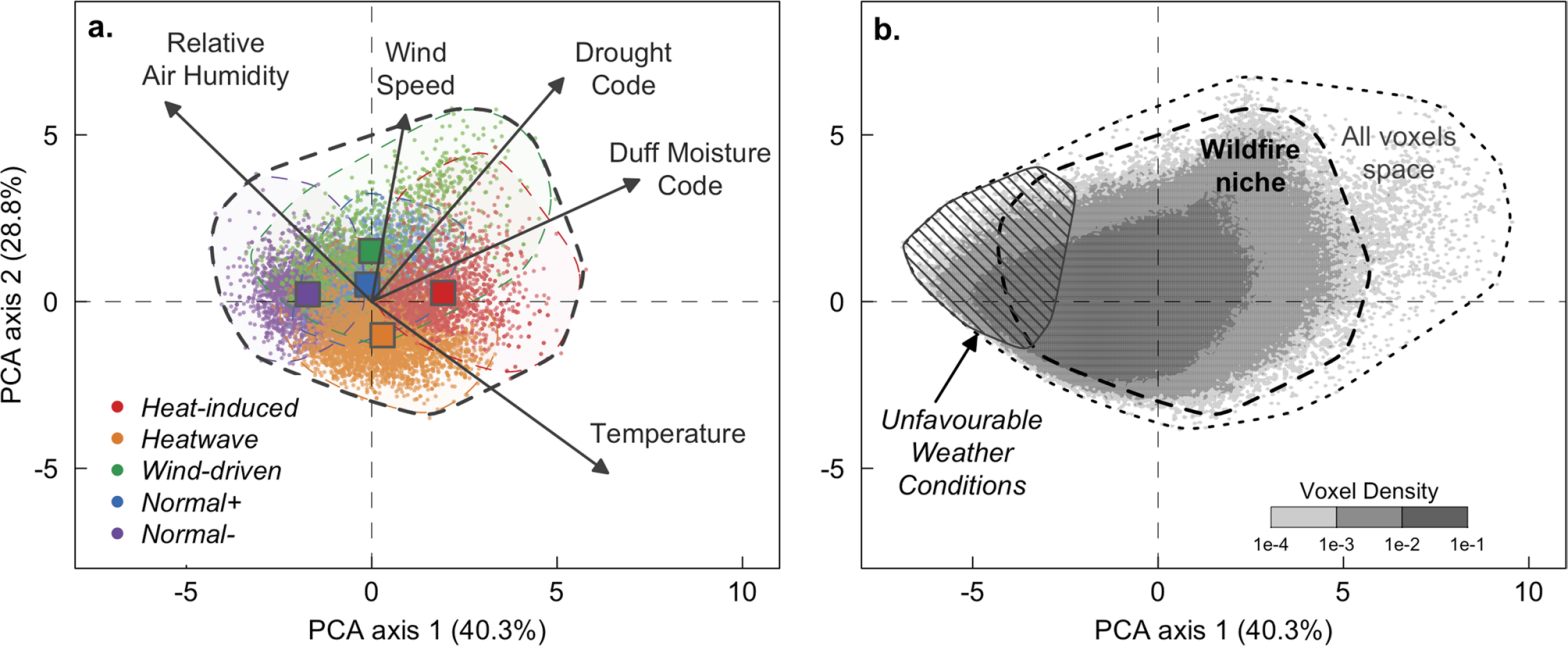
Fire-weather types (FWTs) in the wildfire niche shown in terms of the first two orthogonal combinations of weather and climate variables determined by principal component analysis (PCA). Wildfire records (wildfires > 30 ha) were extracted for the period 1985–2015 from four countries covering most of the biogeographical conditions found in the Mediterranean Basin (France, Greece, Portugal and Tunisia). (**a**) Local daily values of the weather and climate variables associated with each wildfire (colored dots) and centroids (colored squares) for each FWT. (**b**) The wildfire niche is the atmospheric space that contains all the wildfires in the dataset, the *All Voxels* space covers all days and grid cells (including non-fire voxels) in the summer fire season. The *Unfavourable* weather conditions correspond to voxels in which wildfires are unlikely to occur as conditions are moister and cooler than those observed in the wildfire niche.

The set of weather and climate conditions under which wildfires occur (the “wildfire niche”; Fig. 1a) was described by five variables that characterize different levels of fuel aridity (from weekly to monthly scale) and the synchronous, short-term weather conditions (i.e. daily scale) that control the occurrence and spread of wildfires. The variables include the daily mean temperature, wind speed, relative humidity, and two cumulative indices of fuel aridity, namely, the duff moisture code (DMC) and the drought code (DC) of the Canadian Forest Fire Danger Rating System^20^. Daily DMC and DC reflect drought conditions of the past few weeks (DMC) and months (DC), respectively. DMC is generally associated with the moisture content of slow drying surface fuels. The DC was initially developed to estimate the water content of relatively deep and compact duff, and is related in the Mediterranean to long-term adjustments of the living shoots of plants^21^. All meteorological data were extracted from the ECMWF ERA-Interim reanalysis^22^ from 1985 to 2015 at 0.75° spatial resolution across the Mediterranean Basin (details in “Methods”).

**Figure 2:**
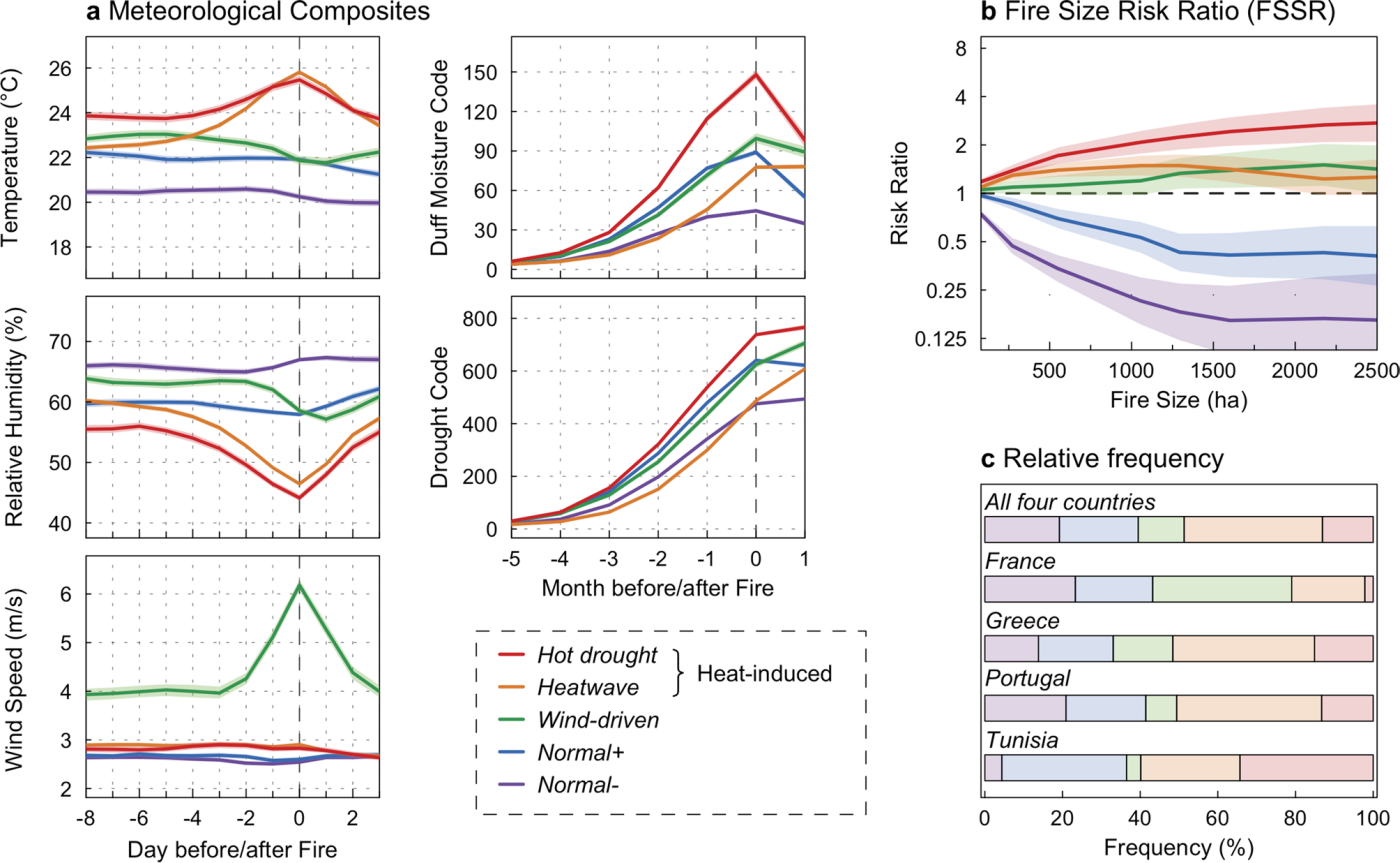
Fire weather type (FWT) characteristics. (**a**) Lead lag composites of weather and climate variables with respect to wildfire days on daily (for temperature, mean relative humidity and mean wind speed) and monthly (for drought code and duff moisture code) timescales. For each FWT, the mean values (lines) and the 95% confidence interval around the mean (colored areas) are reported. The dashed grey vertical lines indicate the day (month) of a fire. (**b**) The Fire Size Risk Ratio (FSRR) for each FWT: mean values (lines) and 95% confidence intervals (colored areas). The FSRR is the ratio of the probability of a wildfire reaching a certain size under a given FWT divided by the corresponding probability under the other FWTs. (**c**) Relative frequency of the FWTs in the four Mediterranean countries investigated here.

Each wildfire in the database was then related to the daily values of the 5 variables observed at the closest ERA-Interim 0.75° grid cell. The weather and climate conditions associated with wildfires were classified into five FWTs through a cluster analysis of the raw variables^17^ (details in “Methods”). Then, we computed for each FWT, the lead lag composites of climate and weather variables on daily (from 8 days before to 2 days after the start of a wildfire for temperature, relative humidity and wind speed) and monthly (from 5 months before to 1 month after the start for DC and DMC) time scales (Fig. 2a) in order to assess the role of instantaneous and short-term weather (i.e. daily atmospheric variables) versus antecedent long-term climate (i.e. monthly DC and DMC) conditions^23^.

Figure 1a shows how FWTs are distributed over the broad range of weather and climate conditions observed during wildfires across the Mediterranean Basin. The five FWTs were named according to their main characteristics and grouped into broader categories to ease their description.

The first category consists of two FWTs, *Normal− *and *Normal*+ (accounting for 19.2 and 20.3% of the wildfires, respectively; Fig. 2c) characterized by little or no change in temperature, humidity or wind speed during the day of the wildfire compared with the preceding or following days (Fig. 2a). This suggests that a number of wildfires are associated with usual weather and climate conditions (Supplementary Figs. S5, S6). The main difference between *Normal−* and *Normal*+ is that the former is significantly moister, with DC and DMC levels below all others FWTs (Fig. 2a). Accordingly, wildfires that occur under the *Normal−* FWT (“*Normal−* wildfires” in the following) occur more frequently in the northern part of the Mediterranean Basin (Fig. 2c) and tend to occur sooner during the fire season than *Normal*+ wildfires do (Supplementary Fig. S4). It should be noted however that the level of droughts observed for the *Normal−* FWT remains, except in Tunisia, in the average of observed summer conditions (Supplementary Figs. S5, S6). These results suggest indirectly that seasonal wildfire activity depends more on fuel aridity in the moister northern part of the Mediterranean Basin^4,24,25^, and more on meteorological conditions associated with influxes of dry, hot air in the drier southern part.

The two heat-induced types of fire-weather, *Heatwave* (35.6% of the wildfires) and *Hot Drought* (13.1% of the wildfires*)* FWTs, are both characterized by drought conditions (higher DC and DMC than the averaged summer conditions) with anomalously higher temperature and lower relative humidity on the day of the wildfire compared with the preceding and following days (Fig. 2a, Supplementary Figs. S5, S6). The temperature and relative humidity on wildfire days under these FWTs are similar, but *Hot Drought* conditions are the driest, with levels of DC and DMC higher than all other FWTs (Fig. 2a). *Hot Drought* wildfires occur more frequently in the southern part of the Mediterranean Basin (Fig. 2c) and tend to occur later in the fire season than *Heatwave* wildfires do (Supplementary Fig. S4).

The last FWT identified in this analysis accounts for 11.8% of wildfires (Fig. 2c) and is induced by the combination of strong winds on the day of the wildfire with relatively dry conditions (Fig. 2a). These *wind-driven* wildfires occur mainly in coastal areas, notably Southern France and Greece (Fig. 2c), where wildfires are often driven by continental northerly synoptic winds respectively known as the Mistral^5^ and Etesian winds^26^.

### Wildfire size under FWTs

In order to evaluate how wildfire size varies according to FWTs, we calculated the Fire Size Risk Ratio (FSRR) for each FWT and for different fire size thresholds (ranging from 80 up to 2,150 ha). FSRR was defined as the probability that a wildfire reaches a given size under a given FWT, normalized by the mean probability for other FWTs (see details in “Methods”). For example, an FSRR of 2 for the 1,000-ha exceedance threshold means that wildfires that ignited under this FWT are twice as likely to exceed 1,000 ha than wildfires occurring under the other FWTs.

Our results show that FSRR is higher when short-term meteorological extremes (warm and dry air, strong winds) combine with long-term summer drought, i.e. under the *Hot drought*, *Heatwave* and *Wind-driven* FWTs (Fig. 2b). Furthermore, among those three FWTs, we observe that FSRR for large to very large wildfire sizes (i.e. > 1,500 ha) is particularly high under *Hot Drought*, thus making compound dry and warm periods as the most favorable conditions for wildfires. By contrast, FSRRs are significantly lower for *Normal−* and *Normal*+ FWTs than for the other FWTs (Fig. 2b), which means that the final size of wildfires that occurred under these FWTs is relatively smaller. We must acknowledge that FSRR vary on more local scales (Supplementary Table S2 in Supplementary material), which highlights that different fuel characteristics, fire management policies or practices are also shaping wildfire size under FWTs.

### Future FWT frequencies in the Mediterranean Basin

The fire-weather classification was applied to current and future summer conditions (June–September) throughout the Mediterranean Basin (i.e. to all gridcell*days independently of the occurrence of wildfires). We used simulations from eight climate models of the EURO-CORDEX initiative^27^, considering the RCP4.5 and RCP8.5 emission scenarios (Supplementary Table S1). To extend the fire-weather classification to summer conditions during which wildfire are unlikely to occur, *i.e*. when fuel moisture prevents significant wildfire spread^13^, the additional type *Unfavourable* was added (Fig. 1b, details in “Methods”).

**Figure 3:**
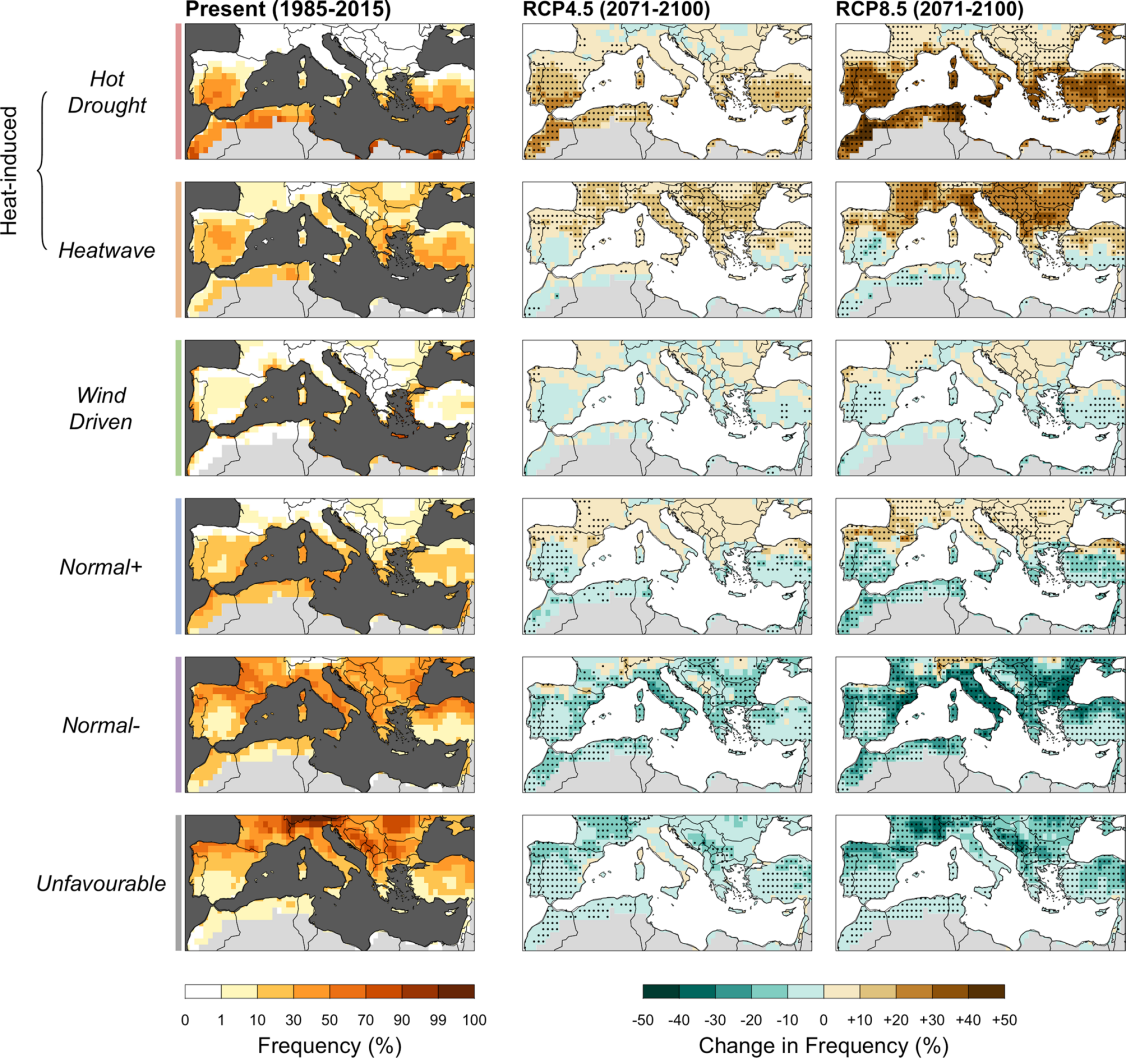
Current and multi-model median changes in summer frequency of fire weather types (FWTs) in the Mediterranean Basin. Changes in frequency at the end of the century (2071–2100) are shown relative to the present period (1985–2015) for two emission scenarios (RCP4.5 and RCP8.5). The dots indicate grid cells where the change was statistically significant (*p* < 0.05) in a majority of the models. The maps were produced in R v3.6.3 (www.r-project.org).

This analysis reveals large shifts in the frequency of FWTs by the end of the century (Fig. 3, right columns). The results are described here only for the 2071–2100 period (with respect to the current climate) under the two concentration pathways, as mid-century (2031–2060) FWT frequencies under both scenarios are similar to the end-of-century frequencies under the RCP4.5 scenario (Fig. 3; Supplementary Fig. S7). The relative frequency of heat-induced FWTs are expected to increase on average by 14% by the end of twenty-first century under the RCP4.5 scenario and by 30% under RCP8.5, with good agreement across models (Supplementary Fig. S8). The projected changes in the relative frequencies of the FWTs differ markedly either side of the 40th parallel (Fig. 3). On the northern side, *Unfavourable* and *Normal− conditions* will become less common while *Normal*+, *Heatwave* and, to a lesser extent, *Hot-Drought* FWTs will increase accordingly. In the drier southern part (Supplementary Fig. S3), our results show that *Hot-Drought* conditions will increase in frequency at the expense of *Normal−* and *Normal*+ FWTs. Our study also demonstrates an increase in the intensity of *Hot-drought* and *Heatwave* FWTs (Supplementary Fig. S9) throughout the Mediterranean Basin (Supplementary Fig. S10). Uncertainty remains, however, as to the extrapolation of FWTs outside the current wildfire niche (Fig. 1b). Under the RCP8.5 scenario, an average of 8.5% of grid-cell*days fall outside the current wildfire niche by the end of the century. Here, these grid-cell*days were related to the closest FWT centroid, while being aware that this hypothesis (conservative) might hide other fire-weather conditions that are not captured by the FWTs defined from present-day climate conditions.

Finally, to assess the potential impact of FWTs shifts on wildfire frequency, we determine the future wildfire frequency in the four countries for which fire datasets were available under the strong hypothesis that the ratio between wildfire frequency and FWT frequency would remain constant in the future (stationarity of the fire-FWT relationship). Our results show that changes in FWT frequency are expected to increase the number of wildfires by 91% in France, 29% in Greece, 21% in Portugal and 30% in Tunisia by the end of the century under the RCP8.5 scenario (Supplementary Figs. S11, S12).

## Discussion

Fire-weather types (FWTs) offer new insights into the climate-induced impacts on wildfire activity as they put into perspective the underlying multi-scale combinations of weather and climate conditions that are conducive to wildfires. While breaking with most studies that investigate wildfire–weather–climate relationships at the eco-regional level^3,4^, our approach provides a complementary and holistic perspective on wildfire–weather–climate relationships where FWTs are the basic unit of analysis. The FWT framework is relevant to projections purposes since it takes full advantage of the regional-scale variability in fire weather and acknowledges that some areas might experience new future fire-weather conditions that have already been experienced in other places. By doing so, we do not intend to ignore or minimize the importance of other factors such as human activities and fuel attributes. Further studies are needed to elucidate how these factors might alter the wildfire-FWT relationships. It is important to note, however, that pooling together four countries with very different socio-economic conditions had limited impact on the characteristics of FWTs (Supplementary Fig. S14), thus demonstrating their robustness.

While substantial disparities in fire–climate relationships have been reported across the Mediterranean Basin^14,17^, our fire-weather based classification highlights the fact that wildfires tend to grow larger when short-term fire–weather conditions co-occur with long-term summer drought. Strong surface winds coupled with low relative humidity and drought conditions are known to favor large wildfires worldwide^5,16^. However, the most extreme wildfires in the Mediterranean Basin mostly occur under *Hot Drought* conditions, i.e., during compound drought and heatwaves. This conclusion matches previous observations on the prevalence of extreme wildfires during heatwaves in the Mediterranean Basin^9,17,28,29,30^. The mechanisms underlying these wildfires remain unclear but different studies have proposed that high temperature and vapor-pressure deficit during heatwaves can accelerate plant desiccation and mortality over relatively short time spans^31–34^, a phenomenon that is expected to be amplified under climate change^34,35^ due the increased frequency and persistence of compound dry-warm periods^36^. Likewise, our results show that future scenarios of fire danger consistently point to an increase in the frequency and intensity of heat-induced FWTs across the Mediterranean. Given that large wildfires generally develop under heat-induced FWTs, climate change might therefore increase their frequency and extent, assuming that fuel remains abundant in spite of increasing aridity. The ecological and socio-economic implications of these changes could be profound, especially in view of the other emerging and interconnected risks that are projected to affect the Mediterranean Basin in the coming decades^37^. To mitigate the risks associated with heat-induced wildfires, dedicated efforts should be made to address current knowledge gaps on the desiccation of live fuels during hot droughts^32^ and the effects of fuel moisture content on wildfire behavior^38^.

However, much uncertainty remains as to what will be the impacts of increased heat-induced FWT frequency on actual wildfire activity. Quantifying wildfire activity is challenging given that several biophysical and human variables influence wildfire occurrence and spread in many and opposite ways. Thus, the expected increase in wildfire activity can be considerably reduced through improvements in suppression capacities and fuel management^39^. Likewise, in the most arid regions of southern Europe, fuel load and fuel continuity may be insufficient to sustain more frequent wildfires^40^, which may challenge the current FWT approach. Conversely, increases to fire ignition in wildland–urban interfaces^41^ or in landscape building of fuels due to systematic fire suppression^42,43^ could further exacerbate the impacts of climate change on wildfire activity.

## Methods

We extracted the location, date, and size of wildfires that occurred in Southern France, Greece, Portugal, and Tunisia (Supplementary Fig. S1) between 1985 and 2015 from national fire databases. To limit uncertainties related to the detection rate of the smallest wildfires and increase the fire-weather signal, wildfires smaller than 30-ha size were excluded. Only summer wildfires (from June to September) were analyzed because 84% of wildfires occurred during these months and accounted for more than 82% of annual burnt area (including of all wildfire sizes, Supplementary Fig. S2). The four countries were selected for two main reasons: (1) the availability of a database with daily information on the characteristics, including the location and size, of all fire events; and (2) the fact that together, they cover most of the biogeographic and socio-economic conditions found in the Mediterranean Basin^44^ (Supplementary Figs. S1–S3). For Southern France, fire statistics were extracted from the “Prométhée” database, managed by French forest services, and examined extensively in previous studies^45,46^. For Greece, fire data from before 1998 were obtained from the Greek forest service and from 2000 onwards, the data were obtained from the Greek fire brigade^47^, such that data for the years 1998 and 1999 are missing. While this combination of sources may have led to some minor inconsistencies, the potential consequences on the results were assumed to be negligible, as year of occurrence was not a factor in the analysis. Fire statistics for Portugal were extracted from the Portuguese rural fire database^48^. For Tunisia, fire statistics were obtained from the Tunisian fire database^49^ as compiled from the records of the Tunisian Directorate-General for Forests and curated from various remote sensing sources. Wildfires from 2011 to 2015 were ignored because of the disruption in fire activity resulting from the Arab spring in December 2010^49^. Fire statistics for the four studied databases are shown in Supplementary Figure S2.

The meteorological data used to determine wildfire variables in the studied period and as a bias correction reference for the climate simulations were taken from the ECMWF ERA-Interim reanalysis^22^ of data from 1985 to 2015 across the Mediterranean Basin (see studied region in Supplementary Fig. S1). To project current and future frequencies of FWTs, we ignored grid cells that contained more than 90% of non-burnable area, as determined from the ESA CCI Land Cover maps for the year 2015 (ESA 2019) at a spatial resolution of 300 m and aggregated at the ERA-INTERIM reference grid cell level. Daily variables were obtained from a 0.75° resolution geographic grid. We used the mean daily 2-m air temperature, dew point temperature, total surface precipitation, surface pressure and 10-m wind speed. Relative humidity was calculated using the mean daily dew point temperature, surface pressure and the mean daily 2-m air temperature.

The DMC and DC subcomponents of the Fire Weather Index^20^ were respectively used as generic indicators of medium and long-term drought. The DMC and DC are calculated in theory from daily 24 h accumulated precipitation data, and the temperature, humidity, and wind speed at 12:00 local time. However, since obtaining modeled data at 12:00 local time was not possible, we used the daily mean temperature, mean humidity and accumulated precipitation. This may have biased the DC and DMC estimates, but time series calculated with both variables are highly correlated^50^. Furthermore, as these fire variables were mostly used for classification purposes, we do not expect this approximation to have a significant impact on the results of the study. Nonetheless, the DC and DMC values calculated here may differ from those computed with the usual 12:00 data.

Projections of weather and climate variables for the current and future climate periods were obtained from two regional climate simulation programs involved in the fifth phase of the Coupled Model Intercomparison Project (CMIP5) and produced as part of the EURO-CORDEX initiative^27,51^. Both include the same eight General Circulation model (GCM)-Regional circulation model (RCM) pairs (involving three RCMs and five GCMs,Supplementary Table S1), but one follows the moderate RCP4.5 scenario while the other follows RCP8.5, i.e. the highest concentration pathway^52^. RCMs were selected based on the availability of daily values for mean temperature, relative humidity, wind speed and precipitation. For each simulation, data were extracted at a spatial resolution of 0.44° in latitude and longitude for the historical (1970–2005) and future (2006–2099) periods in the studied regions (shown in Supplementary Fig. S1). Spatial and seasonal biases in projected climate variables can be problematic for the calculation of fire danger indices^53^ so the data were corrected using univariate and multivariate bias correction based on daily summaries from ECMWF ERA-Interim data for the overlapping period between ECMWF ERA-Interim and climate simulation (1980–2005). The Euro-CORDEX data were interpolated to the regular 0.75° resolution grid of the ERA-INTERIM database using nearest neighbors prior to any other transformation. Statistical corrections were applied on a monthly basis to account for seasonal variations in distributional differences. We used the quantile delta mapping method^54^ for univariate bias correction and the MBCn algorithm^53^ for the multivariate correction. MBCn is a multivariate generalization of quantile mapping that transfers all aspects of an observed continuous multivariate distribution to the corresponding multivariate distribution of variables from a climate model. As both methods yielded similar results (Supplementary Fig. S13), only the results for the univariate biais correction method were used. For each GCM-RCM (Supplementary Table S1), the change in FWT frequencies between the current period (1985–2015) and two future periods (2031–2060 and 2071–2100) were calculated under the RCP4.5 and RCP8.5 concentration pathways.

We identified FWTs objectively by dynamic k-means clustering based on the values of the weather and climate variables associated with each wildfire record, namely, temperature, relative humidity, wind speed, DMC and DC. This method partitions *m* multivariate observations into *k* clusters in which each observation is allocated to the cluster with the nearest mean. In practice, the numerical algorithm minimizes the sum over all clusters of the within-cluster sum of observation-to-centroid squared Euclidean distances. As with any other dynamic clustering method, the value of *k* has to be chosen beforehand. To optimize our understanding of fire-weather relationships at the continent level, *k* was chosen as a trade-off between three competing criteria namely, (1) that *k* should be large enough to discriminate between the maximum possible combinations of wildfire predictors, (2) that *k* should be small enough for the results to be generalizable and to facilitate interpretation, and (3) that the final set of clusters should provide a sound physical interpretation of fire-weather relationships. We therefore set the clustering algorithm to identify *k* = 5 FWTs. The stability of the clusters was verified by rerunning the algorithm with different initial random seeds. Furthermore, since the wildfires were not evenly distributed between the studied countries (Supplementary Fig. S2; Supplementary Table S2), we also tested the stability of the clusters against variations in the proportion of wildfires from each country. The same k-means analysis was performed on 1,000 bootstrap-resampled wildfire datasets, in which the number of wildfires from each country was proportional to the corresponding number of wildfire gridcells. The FWTs in the original and resampled datasets had similar characteristics (Supplementary Fig. S14), confirming the robustness of this approach. For representation purposes, the weather and climate variables were compressed into orthogonal linear combinations using PCA. Only the first two principal components were retained, as they adequately described most (69%) of the variability of the data. All gridcell*day (voxel) combinations (i.e. not just fire*day combinations) were then plotted in PCA space for the four studied countries.

The FWTs were characterized using a lead-lag analysis of composite climate variables. As wildfires in the Mediterranean Basin are short, we focused mainly on pre-ignition conditions. Lead-lag composites were examined over two timescales to capture the seasonal and synoptic variability associated with fire occurrence. We used an 11-day window (from 8 days before to 2 days after the start of a fire) for the meteorological variables (mean temperature, relative humidity and wind speed) and a 7-month window (from 5 months before to 1 month after the start of a fire; usual calendar months) for the fuel aridity variables.

To assess whether wildfires grow larger in size under particular FWTs, we calculated a fire size risk ratio (FSRR) for each FWT. The FSRR of an FWT and a fire size *S* is the ratio of the probability of a fire reaching size *S* in FWT *T* divided by the probability of a fire reaching the same size in the other FWTs. According to this definition, an FSRR higher (alternatively lower) than 1 indicates that a fire of size *S* is more (alternatively less) likely in FWT *T* than in the others. FSRRs were calculated for eight fire sizes (30, 80, 271, 550, 1,055, 1,295, 1,600 and 2,173 ha), corresponding respectively to the 1st, 50th, 80th, 90th, 95th, 96th, 97th and 98th percentiles of the data (Supplementary Table S2).

For each GCM-RCM (Supplementary Table S1), FWT frequencies for the current period (1985–2015) and two future periods (2031–2060 and 2071–2100) were calculated under the RCP4.5 and RCP8.5 concentration pathways for each gridcell. Voxels located outside the wildfire niche in the bottom-left quadrant of the principal component subspace (Fig. 1b) were considered representative of *Unfavourable* weather conditions. This additional FWT consists of all the voxels from the *Normal−* FWT whose Euclidian distance to the centroid was greater than the 95% confidence interval.

## Supplementary information

Supplementary file1
