# Supplementary material for: Increased likelihood of heat-induced large wildfires in the Mediterranean Basin
Source: Sci Rep. 2020 Aug 14;10:13790. doi: 10.1038/s41598-020-70069-z (PMC7427790; doi:10.1038/s41598-020-70069-z)
Supplement: Supplementary file 1 — Supplementary file1 [file 41598_2020_70069_MOESM1_ESM.pdf]

Supplementary material

Increased likelihood of heat-induced large wildfires in the Mediterranean Basin

Julien Ruffault[1,10,*], Thomas Curt[2], Vincent Moron[3], Ricardo M. Trigo[4,5], Florent Mouillot[6], Nikos Koutsias[7], François Pimont[8], Nicolas Martin-StPaul[8], Renaud Barbero[2], Jean-Luc Dupuy[8], Ana Russo[4] Chiraz Belhadj-Khedher[9]

[1] Institut Méditerranéen de Biodiversité et d'Ecologie marine et continentale (IMBE), Aix Marseille Université, CNRS, IRD, Avignon Université, Marseille, France

[2] INRAE, Aix Marseille Univ, RECOVER, Aix-en-Provence, France

[3] Aix Marseille University, CNRS, IRD, INRAE, Coll. de France, CEREGE, Aix-en-Provence, France

[4] Instituto Dom Luiz (IDL), Faculdade de Ciências, Universidade de Lisboa, Campo Grande 1749-016, Lisbon, Portugal

[5] Departamento de Meteorologia, Instituto de Geociências, Universidade Federal do Rio de Janeiro, Rio de Janeiro, 21941-916, Brazil

[6] CEFE, UMR 5175, Univ. Montpellier, CNRS, EPHE, IRD, univ. Paul Valery Montpellier 3, 1919 route de Mende, 34293 Montpellier Cedex 5, France

[7] Department of Environmental Engineering University of Patras G. Seferi 230100, Agrinio, Greece

[8] INRAE, Ecologie des Forêts Méditerranéennes (UR 629), Avignon, France

[9] Geomatics and Geosystems, University of Manouba, Manouba, Tunisia

[10] Present Address : INRAE, Ecologie des Forêts Méditerranéennes (UR 629), Avignon, France

[*] Corresponding author: julien.ruff@gmail.com

ID	Forcing GCM	Run	RCM	Institution
1	CNRM-CM5	r1i1p1	RCA 4	Swedish Meteorological and Hydrological Institute (SMHI)
2	ICHEC-EC-EARTH	r12i1p1	RCA 4	Swedish Meteorological and Hydrological Institute (SMHI)
3	MPI-ESM-LR	r1i1p1	RCA 4	Swedish Meteorological and Hydrological Institute (SMHI)
4	IPSL-CM5A-MR	r1i1p1	RCA 4	Swedish Meteorological and Hydrological Institute (SMHI)
5	MHOC-HadGEM2-ES-01	r1i1p1	RCA 4	Swedish Meteorological and Hydrological Institute (SMHI)
6	ICHEC-EC-EARTH	r1i1p1	RACMO 2.2	Royal Netherlands Meteorological Institute (KNMI)
7	MHOC-HadGEM2-ES-01	r1i1p1	RACMO 2.2	Royal Netherlands Meteorological Institute (KNMI)
8	ICHEC-EC-EARTH	r3i1p1	HIRHAM 5	Danish Meteorological Institute (DMI)

Table S1 EURO-CORDEX experiments used in this study. Each experiment includes one historical and two scenario (RCP4.5 and RCP8.5) runs; spanning the periods 1970-2005 and 2006-2099 respectively.

	Fire weather Type	Final fire size (ha)							
		30	80	271	550	1055	1295	1600	2173
All four countries	*Normal-*	2571	1076	291	108	35	24	16	11
	Normal+	2765	1446	531	222	88	58	42	29
	Wind-driven	1624	911	380	194	103	90	71	50
	Heatwave	4909	2787	1245	649	337	269	196	121
	Hot drought	1815	1122	518	308	179	151	120	86
France	*Normal-*	225	90	34	16	10	8	8	6
	Normal+	206	128	54	24	13	13	12	10
	Wind-driven	378	236	111	64	37	31	24	16
	Heatwave	192	100	55	27	18	13	8	3
	Hot drought	24	12	4	4	3	2	2	2
Greece	*Normal-*	435	145	30	5	2	2	0	-
	Normal+	617	290	101	30	10	9	8	6
	Wind-driven	505	289	131	60	37	32	26	20
	Heatwave	1182	655	282	142	76	64	48	25
	Hot drought	502	295	116	71	36	31	25	19
Portugal	*Normal-*	1907	840	227	87	23	14	8	5
	Normal+	1903	1006	372	165	63	34	21	13
	Wind-driven	737	384	137	69	28	26	19	14
	Heatwave	3504	2011	901	475	242	191	139	92
	Hot drought	1243	783	389	228	138	116	91	63
Tunisia	*Normal-*	4	1	0	0	0	0	0	0
	Normal+	39	22	4	3	2	2	1	0
	Wind-driven	4	2	1	1	1	1	1	0
	Heatwave	31	21	7	5	1	1	1	1
	Hot drought	46	32	9	5	2	2	2	2

Table S2 Wildfire size distribution according to each fire weather type (FWT) for the entire wildfire dataset and for each of the country examined.

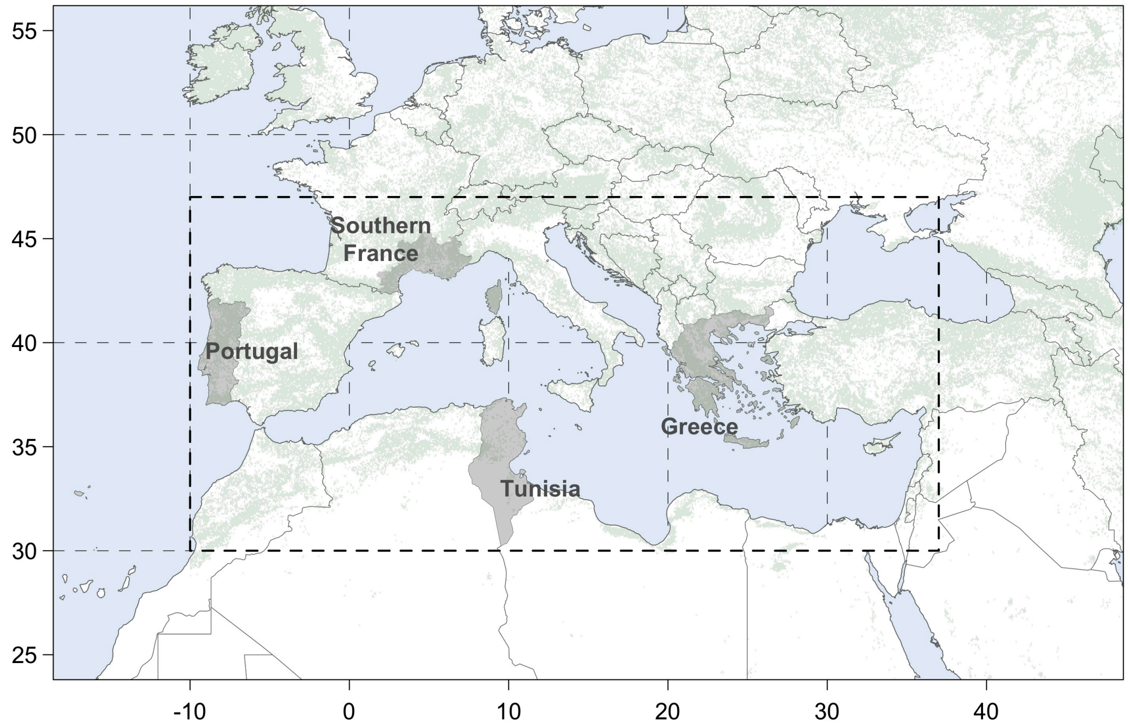

Fig. S1 Location of the four countries for which wildfire records were examined (Southern France, Greece, Portugal, and Tunisia) within the Mediterranean geographic domain considered in this study (dashed rectangle). The map was produced in R v3.6.3 (www.r-project.org).

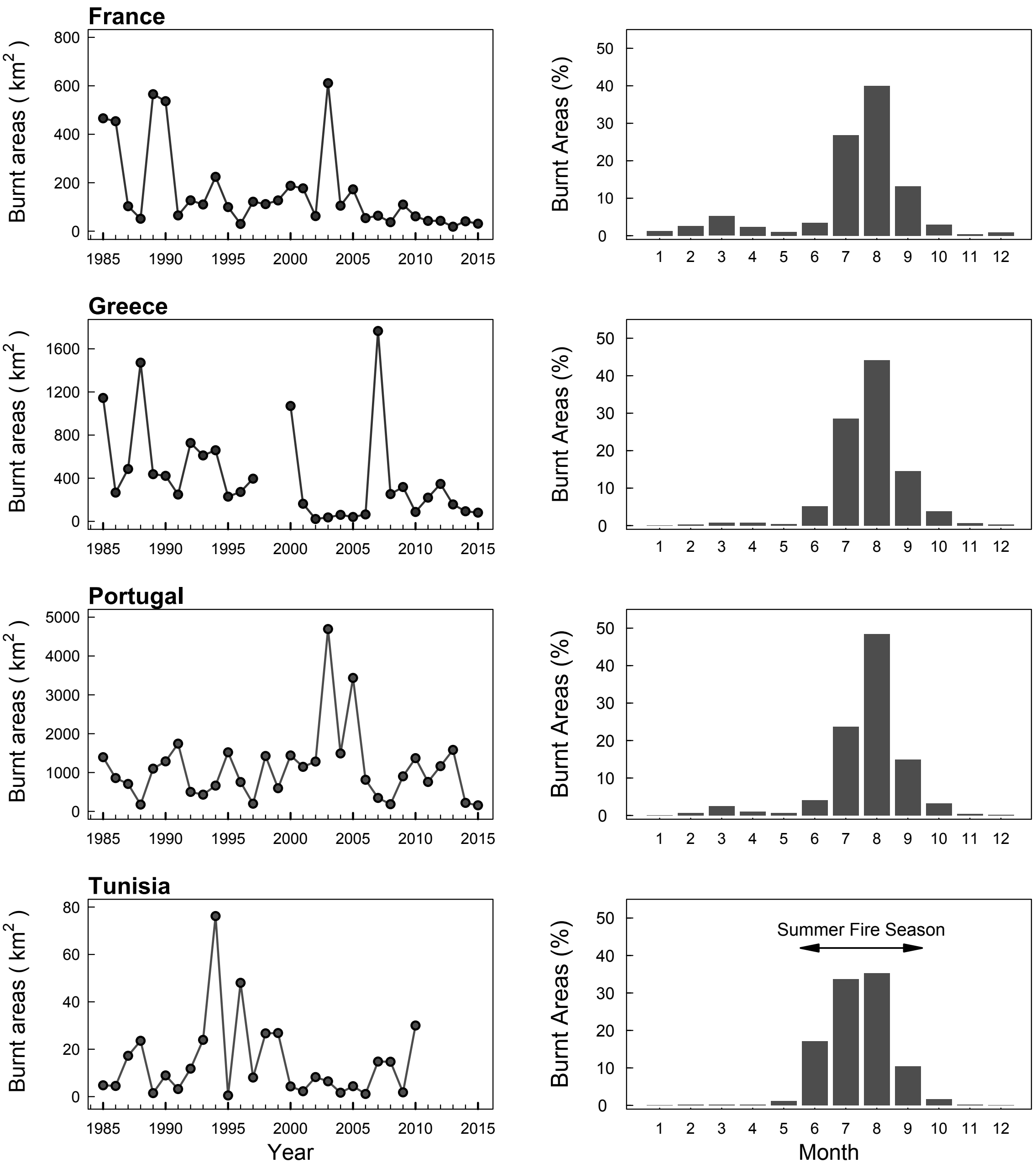

Fig. S2 Interannual and Seasonal variability in wildfire activity for each of the four countries examined. Data is missing for years 1998 and 1999 for Greece and for the period from 2011 to 2015 for Tunisia (see details in methods). The summer wildfire season (June, July, August and September) is indicated.

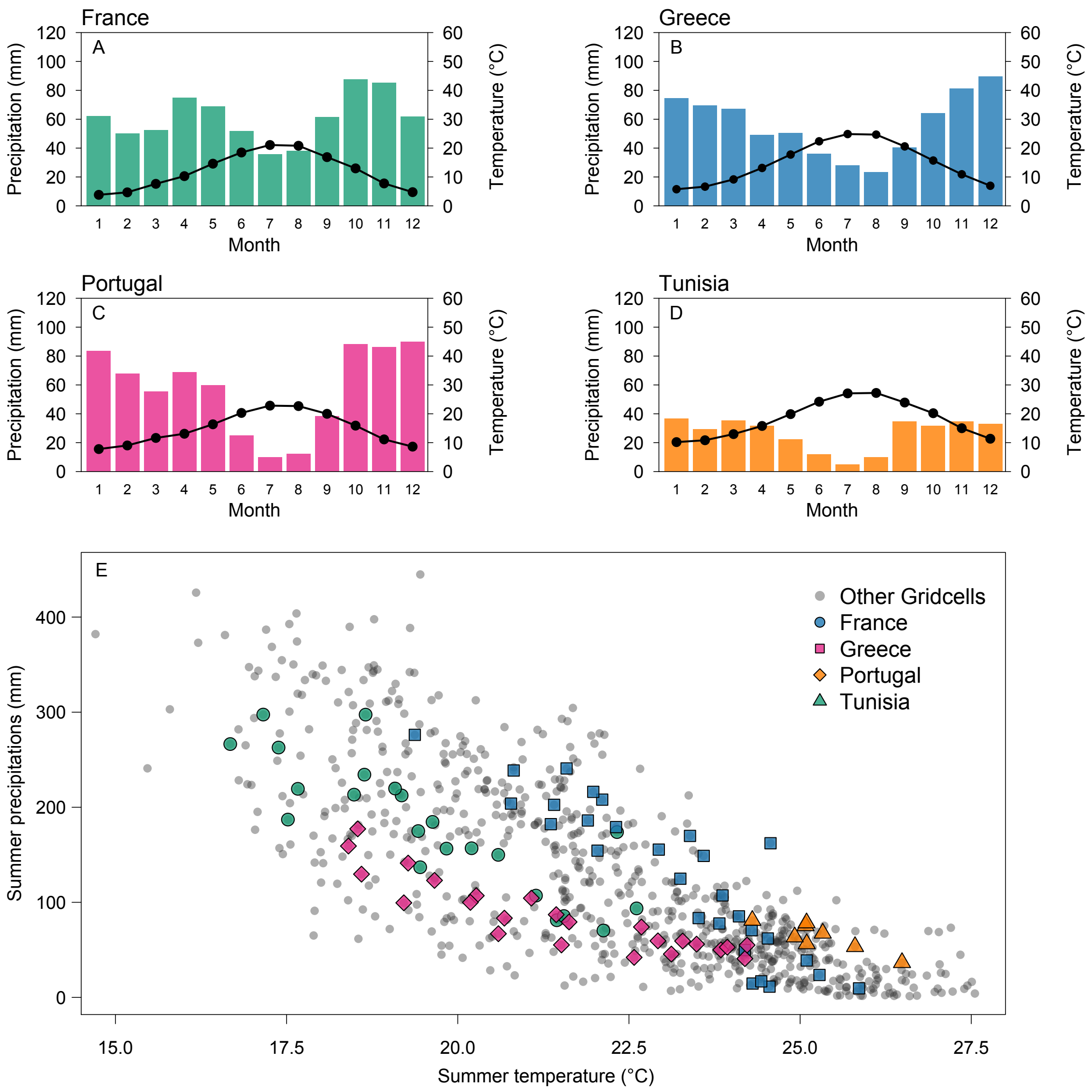

Fig. S3 Mean climatic conditions (1985-2015) in the four countries examined (A, B, C and D) and their comparison to the rest of the Mediterranean basin (E). The four ombrothermic diagrams in A, B, C and D show the differences in summer conditions between the four countries. Subpanel E shows that the four countries examined encompasses most of the variability in mean summer conditions found in the Mediterranean Basin.

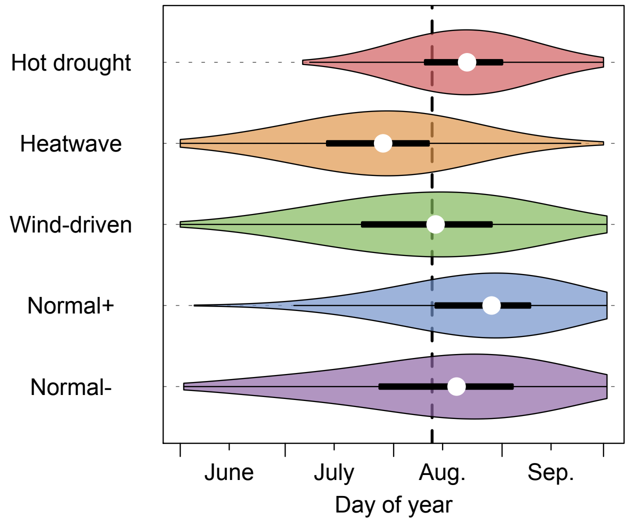

Fig. S4 Seasonality of wildfires (>30 ha) according to each fire weather type (FWT). Violin plots show a kernel density estimation of the distribution of the day of year. White dots indicate the median of the parameter set, black boxes indicate the interquartile range between the 25th and 75th percentile, the thin black lines indicate the upper and lower adjacent values. Dashed black vertical lines denote the median for all fires. Medians for all FWTs are statistically different from each other ($p<0.001$, Pairwise Wilcoxon tests with Bonferroni correction).

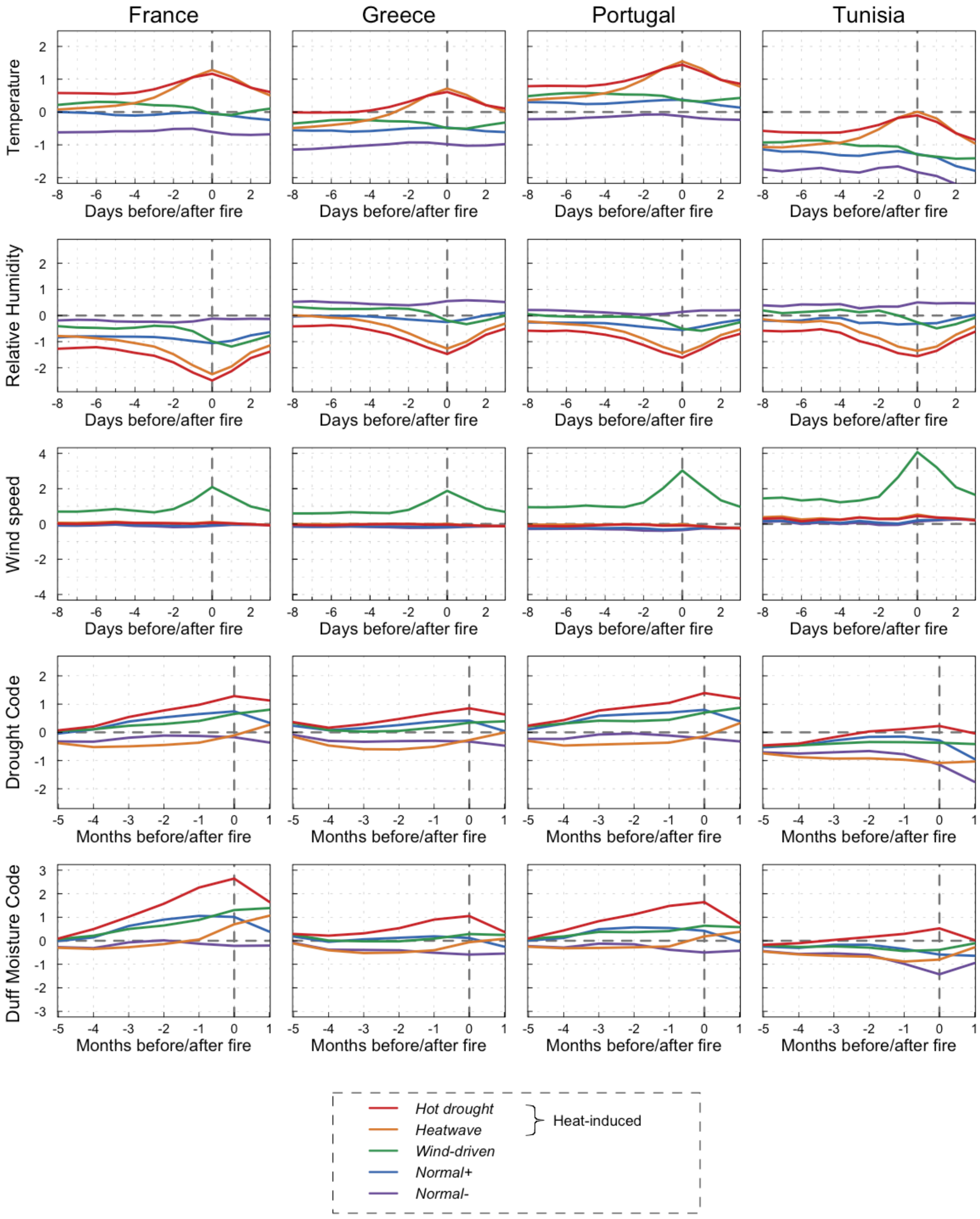

Fig. S5 Composites of standardized anomalies associated with the different fire weather types (FWTs) relative to reference climates for each of the four countries examined. Lead lag Composites of standardized anomalies at daily (for temperature, mean relative humidity and mean wind speed) and monthly (drought code and duff moisture code) time scale are indicated.

Dashed grey vertical lines denote the day (or month) of fire. Standardized anomalies were compiled for each country: a reference climate was estimated in each country and the standardized anomalies were calculated in comparison. The reference climates were determined by randomly selecting 10,000 non-fire gridcell*day voxels with the same location in space and time (in the annual cycle) as the fires. This allowed a fair comparison between fire and non-fire atmospheric conditions with the same spatial and temporal frames.

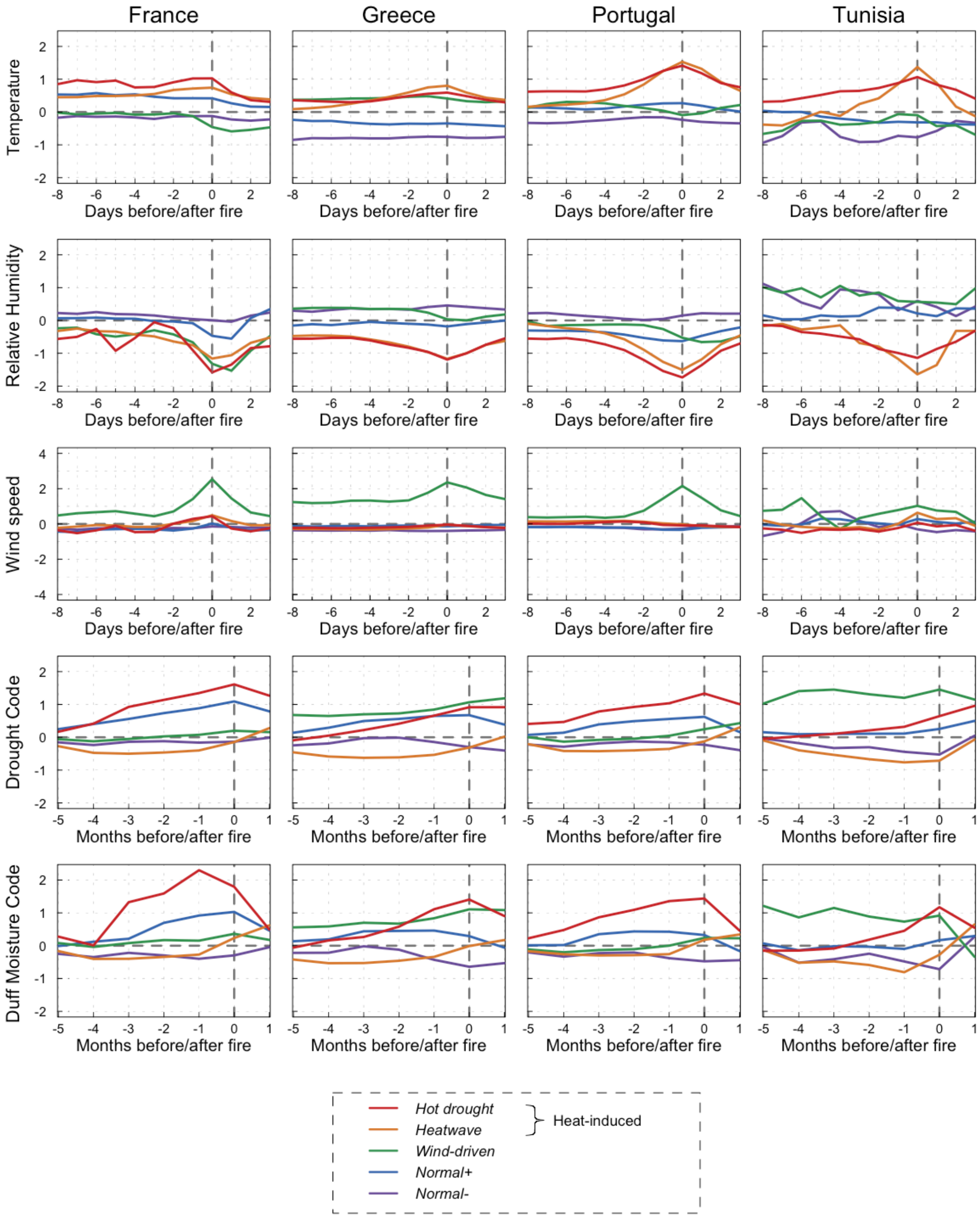

Fig. S6 Same as Fig. S5 but composites were determined for each country only with the wildfires that occurred within that country (not the entire wildfire dataset).

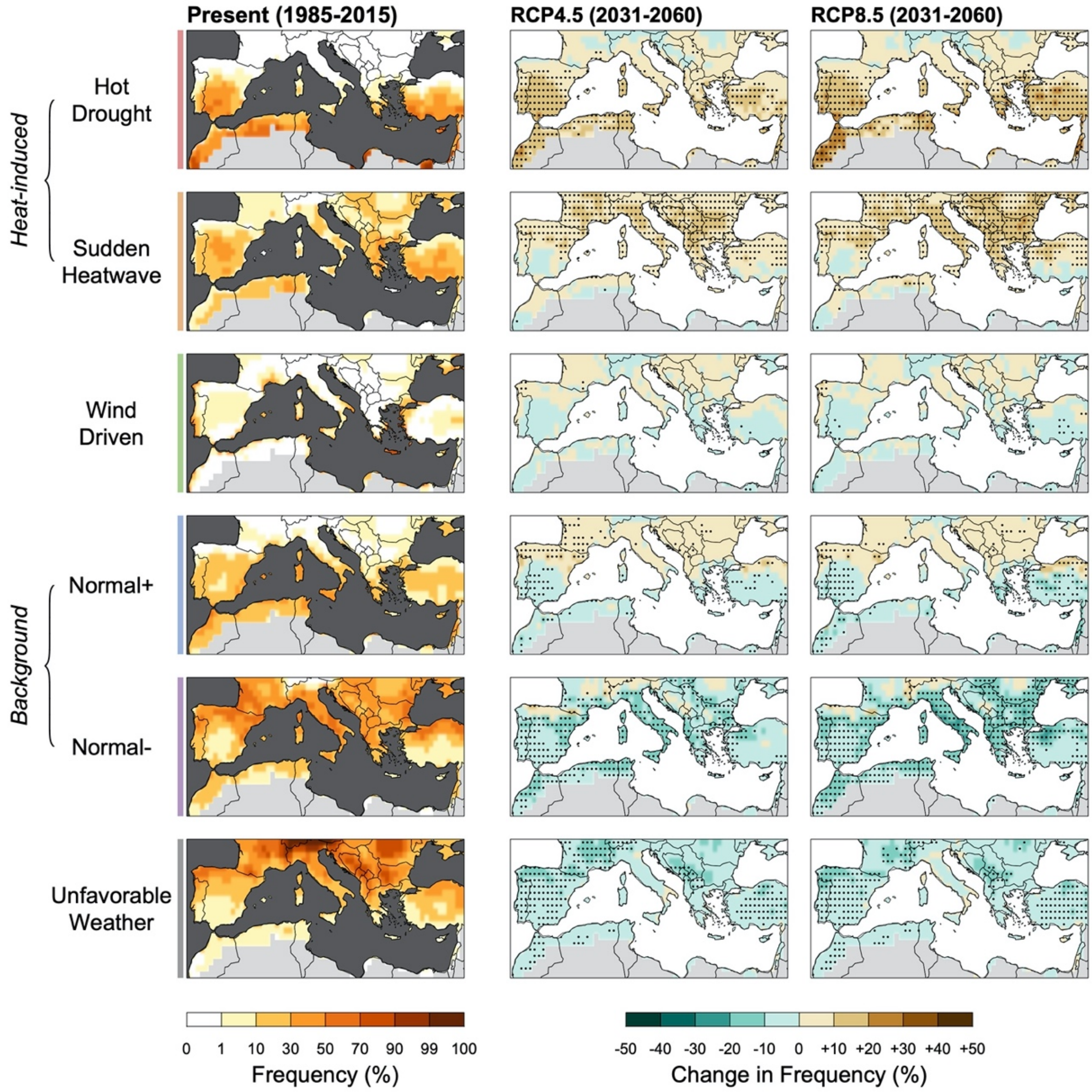

Fig. S7 As in Fig. 3 but for the period from 2031 to 2060. The maps were produced in R v3.6.3 (www.r-project.org).

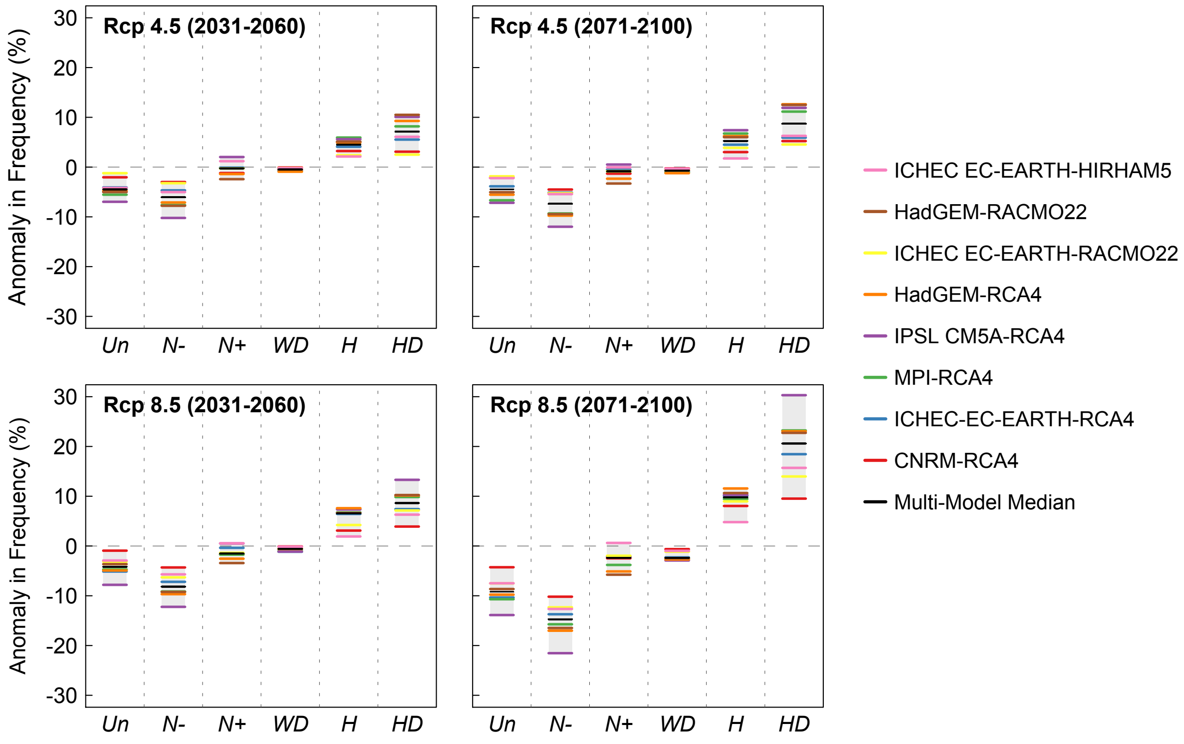

Fig. S8 Changes in the summer frequency of fire weather types (FWTs) between the future (2031-2060; 2071-2100) and present (1985-2015) periods for two scenarios (RCP4.5 and RCP8.5) averaged over the Mediterranean Basin. The 8 GCM-RCM examined (colored dashes) and the multi-model median (black dashes) are indicated. Dashed grey horizontal lines denote zero change in frequency. *Unfavourable* (Un) *Normal-* (N-), *Normal+* (N+), *Wind-Driven* (WD), *Heatwave* (H) and *Hot-drought* (HD).

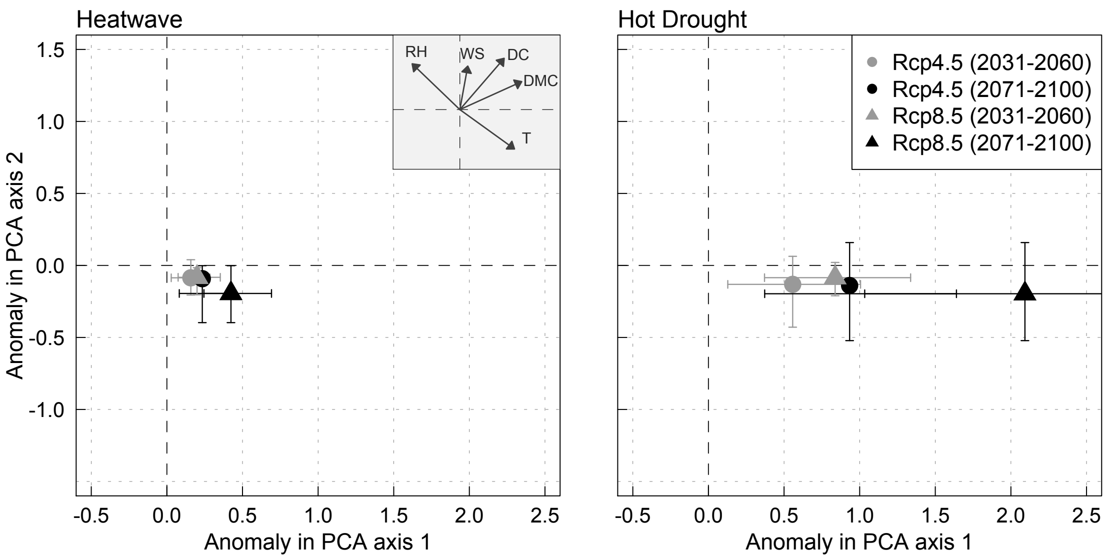

Fig. S9 Multi model median change in the intensity of *n Heatwave* and *Hot-drought* FWTs over the Mediterranean Basin shown in terms of the first two orthogonal combinations of wildfire predictors determined by principal component analysis (PCA) of five daily climate variables driving wildfires. FWT intensity is determined as the centroid of the furthest 10% gridcells*days from the FWT centroid for the entire Mediterranean Basin. Changes are expressed at end of the twenty-first century (2071–2100) relative to the present period (1985-2015) for two scenarios (RCP4.5 and RCP 8.5). Dashed black horizontal lines denote zero change in frequency. Minimum and maximum values for each of the 8 RCM models are indicated by horizontal and vertical arrows. The subpanel on the right panels indicates the loadings of climate variables in the PCA: relative humidity (RH), wind speed (WS), drought code (DC), duff moisture code (DMC) and temperature (T).

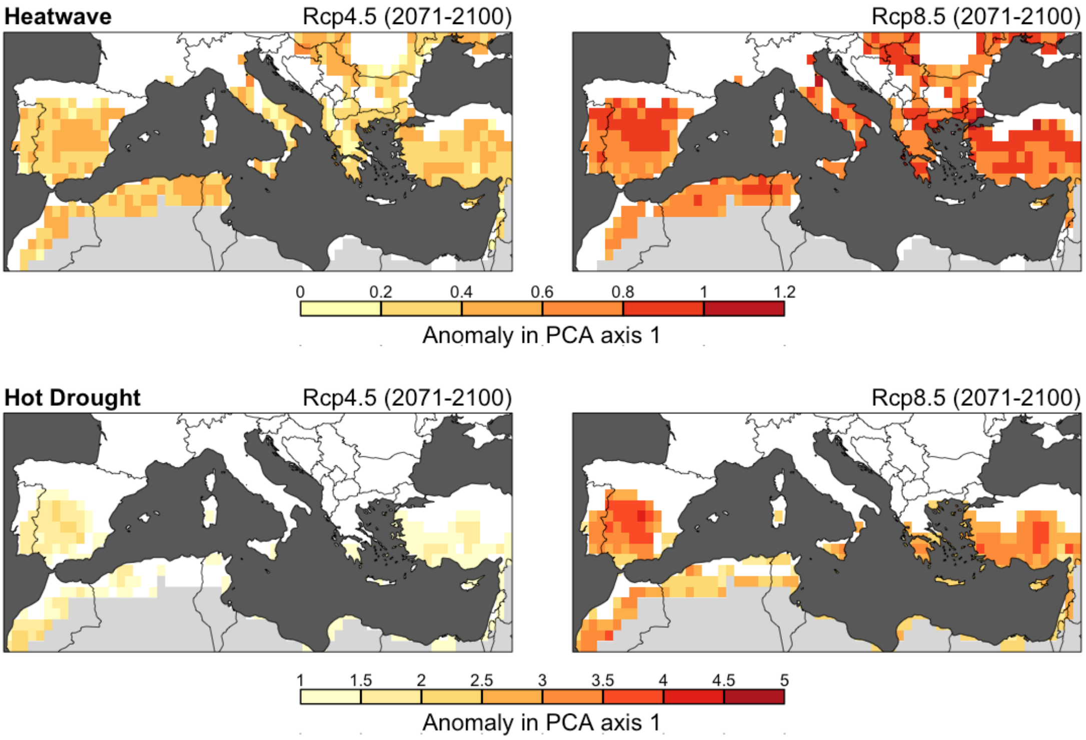

Fig. S10 Multi model median change in the intensity of *Sudden Heatwave* and *Hot-drought* FWTs shown in terms of the first combination of wildfire predictors determined by principal component analysis (PCA) of five daily climate variables driving wildfires. FWT intensity is determined as the centroid of the furthest 10% gridcells*days from the FWT centroid for the entire Mediterranean Basin. Changes are expressed at end of the twenty-first century (2071–2099) relative to the present period (1985-2015) for two forcing scenarios (RCP4.5 and RCP8.5). The maps were produced in R v3.6.3 (www.r-project.org).

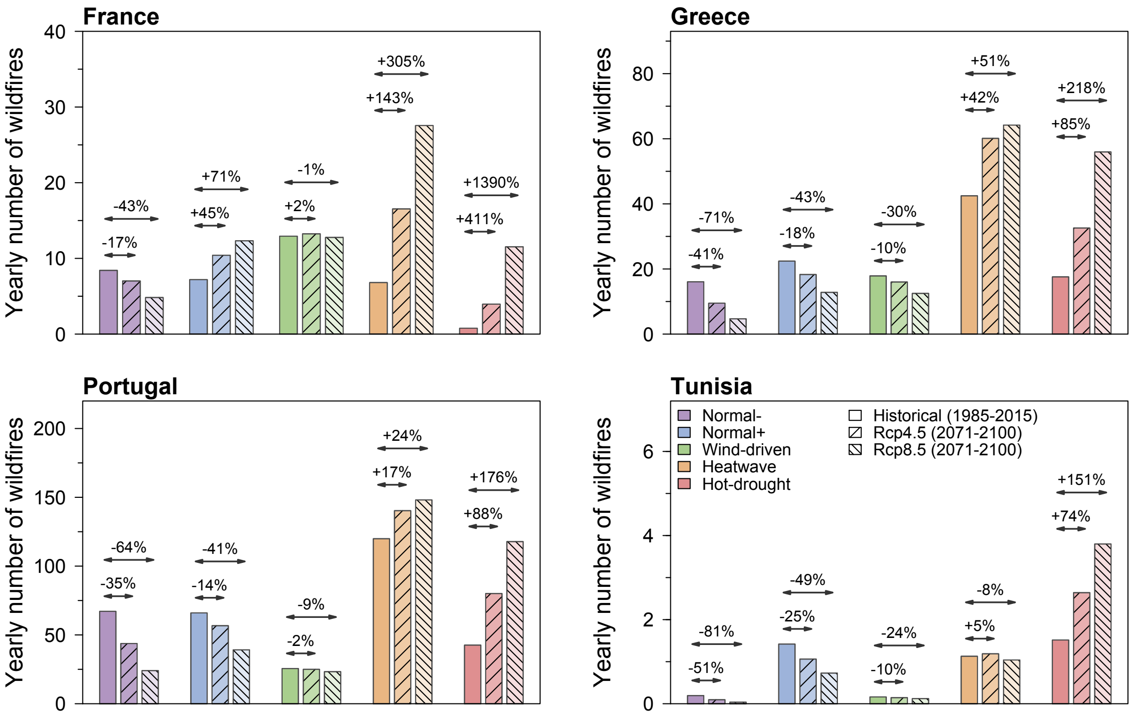

Fig. S11 Observed and projected multi-model median of the yearly number of wildfires (>30 ha) for each fire weather type (FWT) in each of the four countries. Present (1985-2015) and future (2071-2100) periods for two scenarios (RCP4.5 and RCP8.5) are indicated.

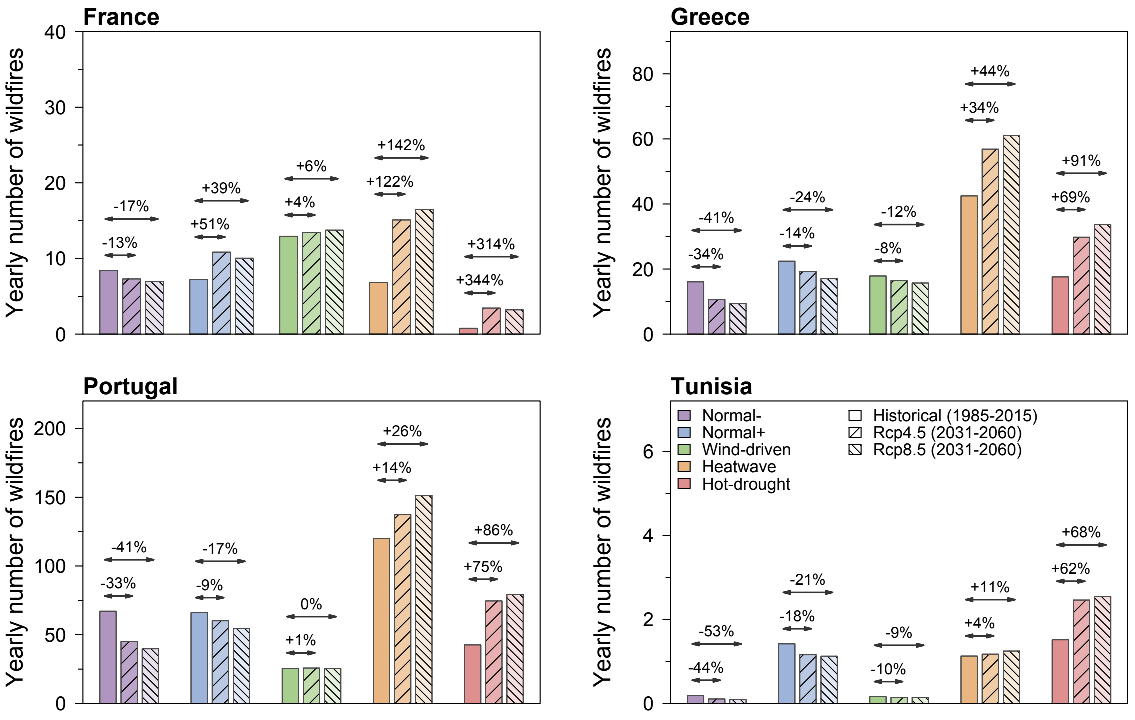

Fig. S12 as Fig. S11 but for the period 2031-2060.

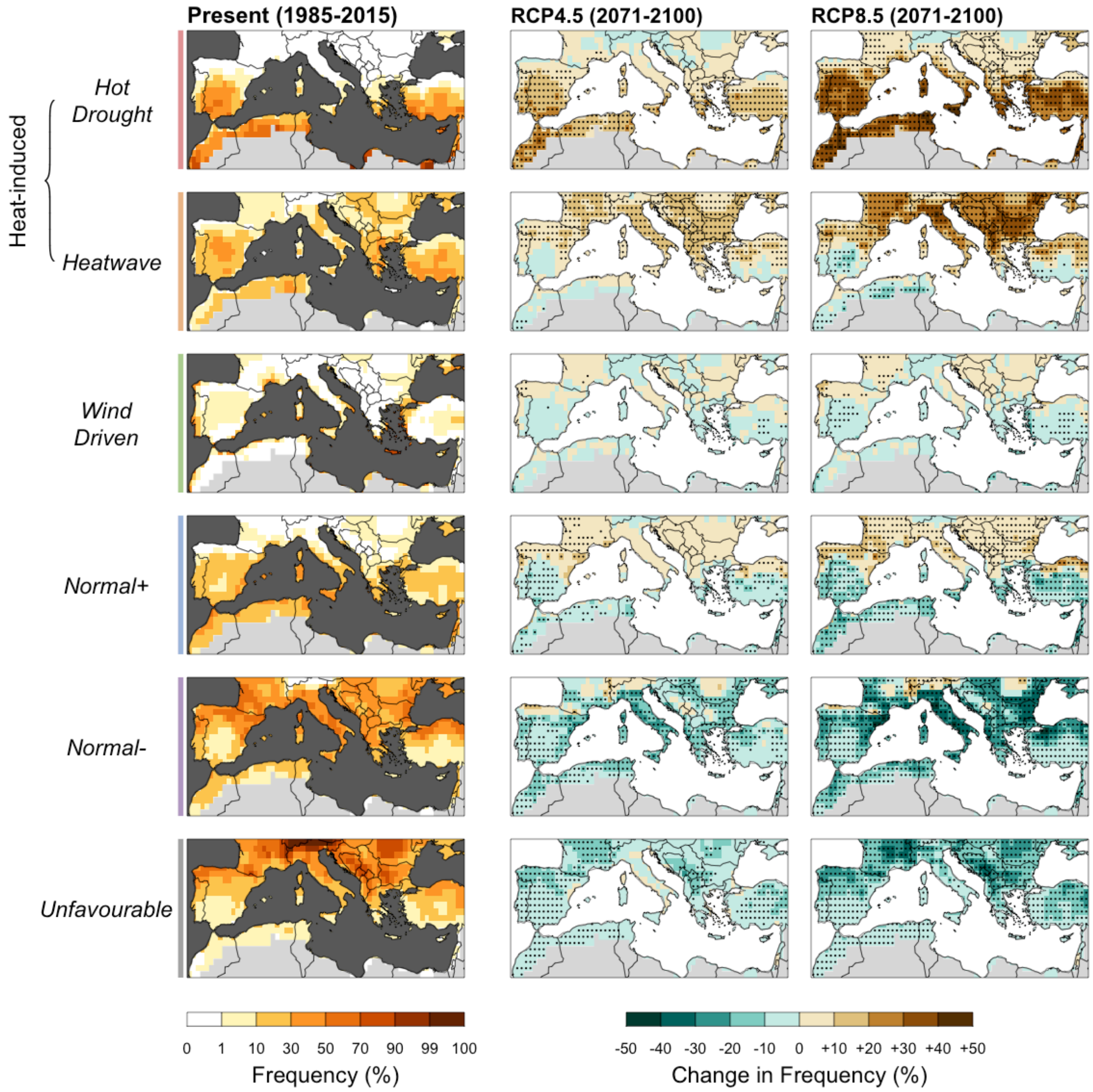

Fig. S13 as Fig. 3 but using multivariate bias correction procedures. The maps were produced in R v3.6.3 (www.r-project.org).

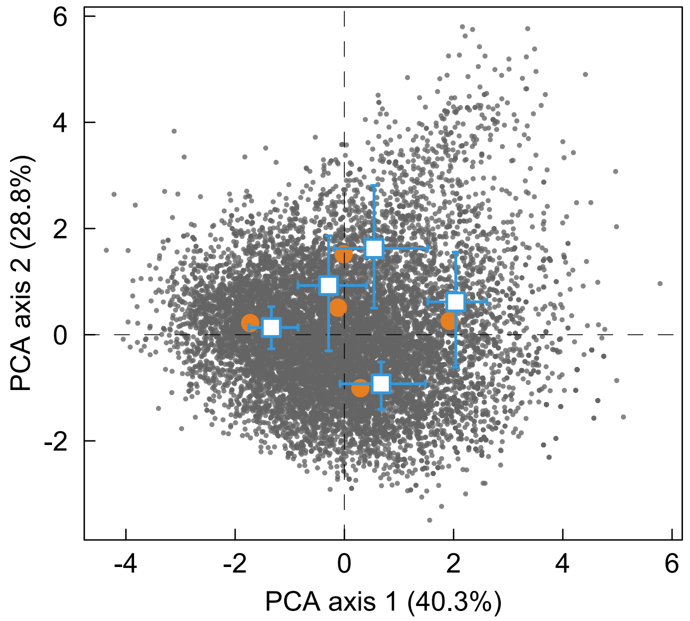

Fig. S14 Stability of the clusters against variations in the proportion of wildfires from each country shown in terms of the first two orthogonal combinations of wildfire predictors determined by principal component analysis (PCA). Blue squares indicate the medians of centroids resulting from the k-means analysis performed on 1,000 bootstrap-resampled wildfire datasets, in which the number of wildfires from each country was proportional to the corresponding number of wildfire gridcells. Error bars show the 5 and 95% confidence intervals. Daily values of the climate variables associated with each wildfire (grey dots) and centroids (orange dots) resulting from the original wildfire dataset (as in Fig. 1a.) are indicated for reference.
